# Supplementary figures and images for: Optimization of Hermetia illucens (L.) egg laying under different nutrition and light conditions
Source: PLoS One. 2020 Apr 24;15(4):e0232144. doi: 10.1371/journal.pone.0232144 (PMC7182258; doi:10.1371/journal.pone.0232144)

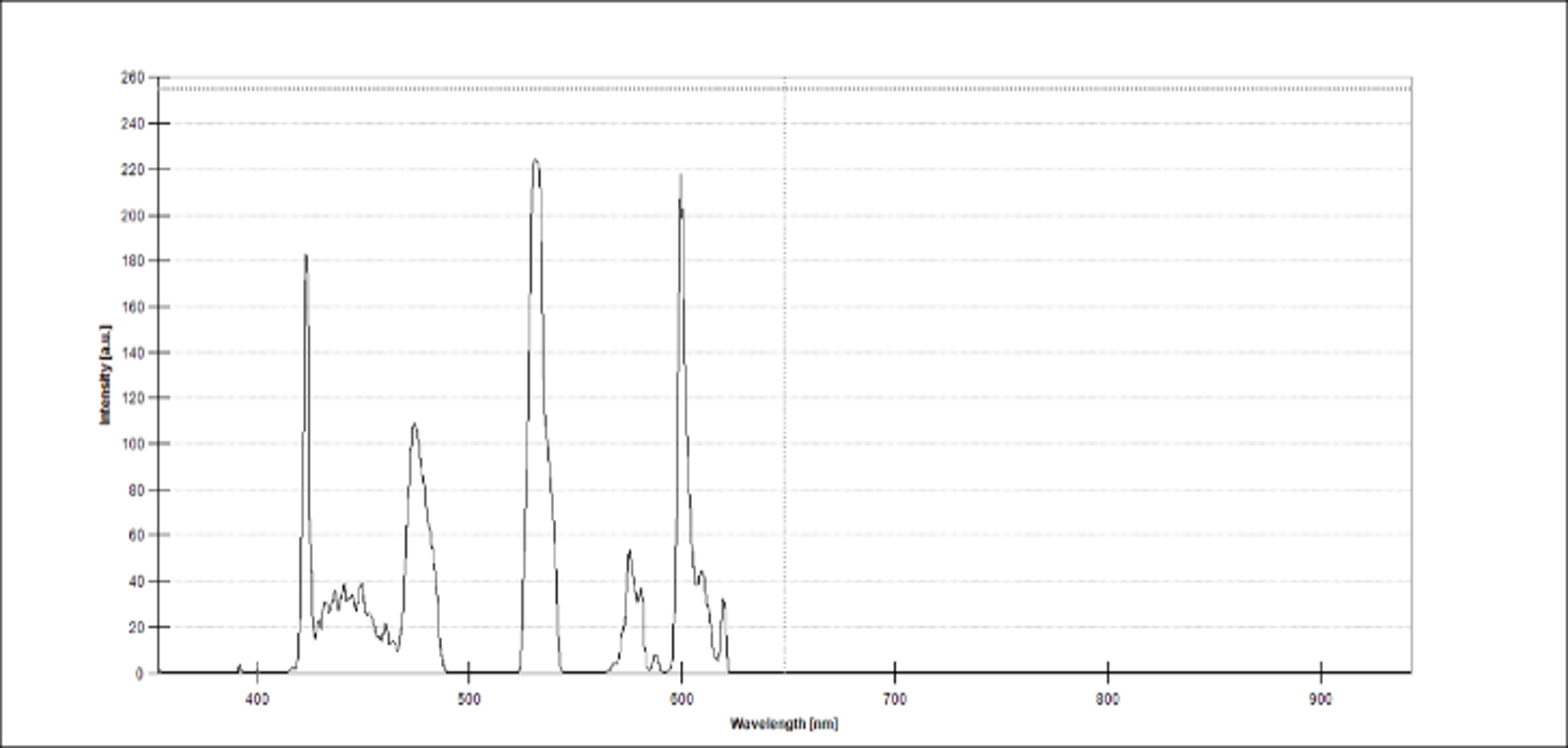

Supplement: S1 Fig — (TIF) [file pone.0232144.s001.tif]

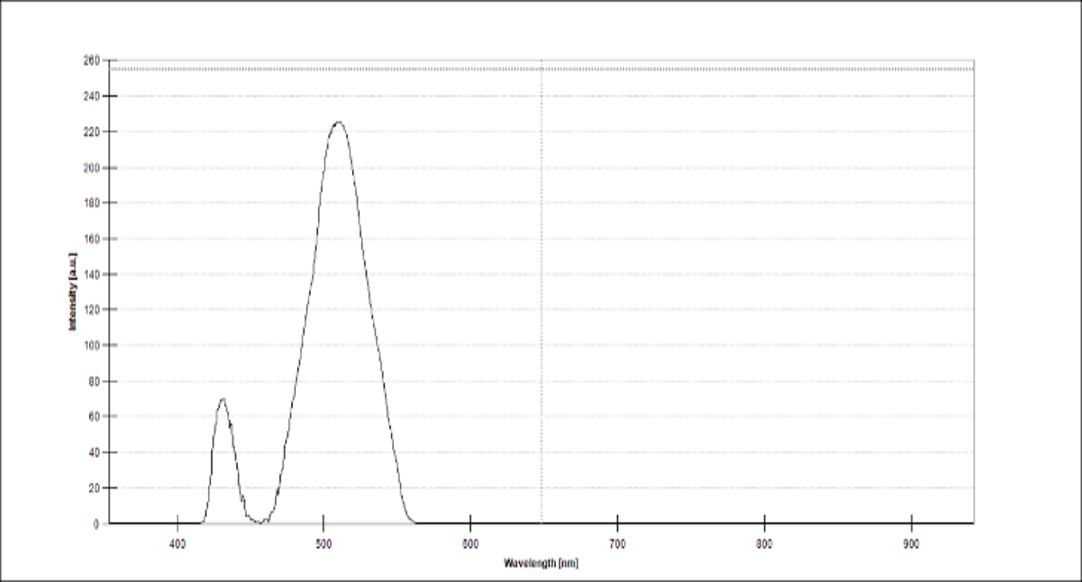

Supplement: S2 Fig — (TIF) [file pone.0232144.s002.tif]

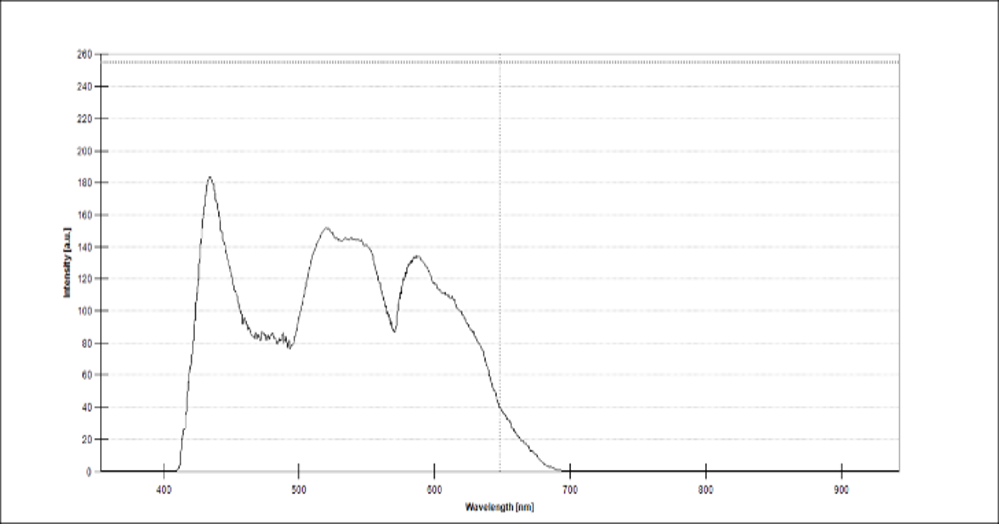

Supplement: S3 Fig — (TIF) [file pone.0232144.s003.tif]

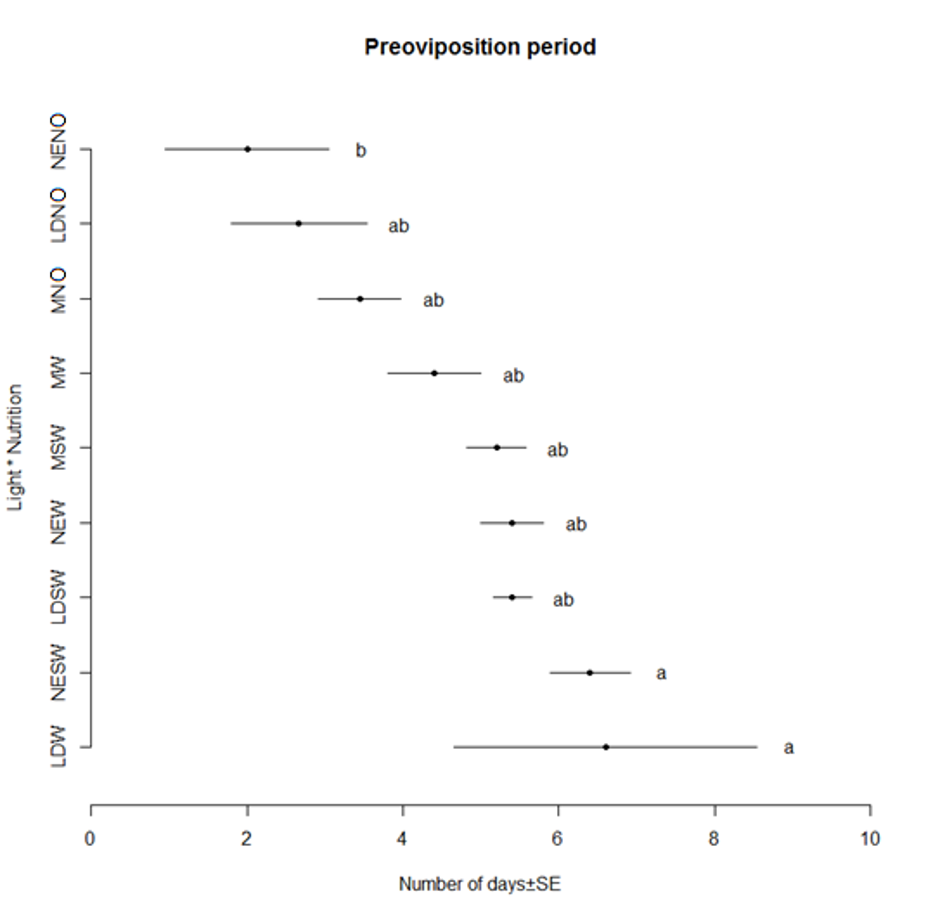

Supplement: S4 Fig — Different letters within each column are significantly different between treatments. (TIF) [file pone.0232144.s004.tif]

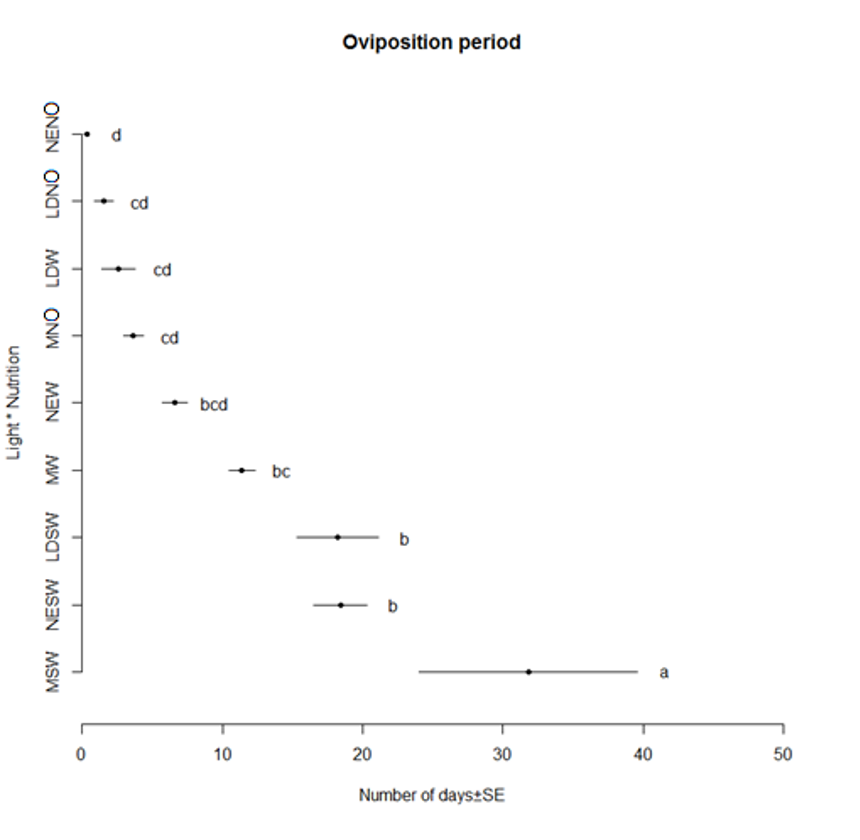

Supplement: S5 Fig — Different letters within each column are significantly different between treatments. (TIF) [file pone.0232144.s005.tif]

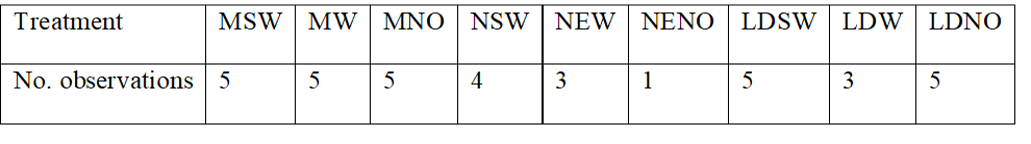

Supplement: S1 Table — (TIF) [file pone.0232144.s006.tif]

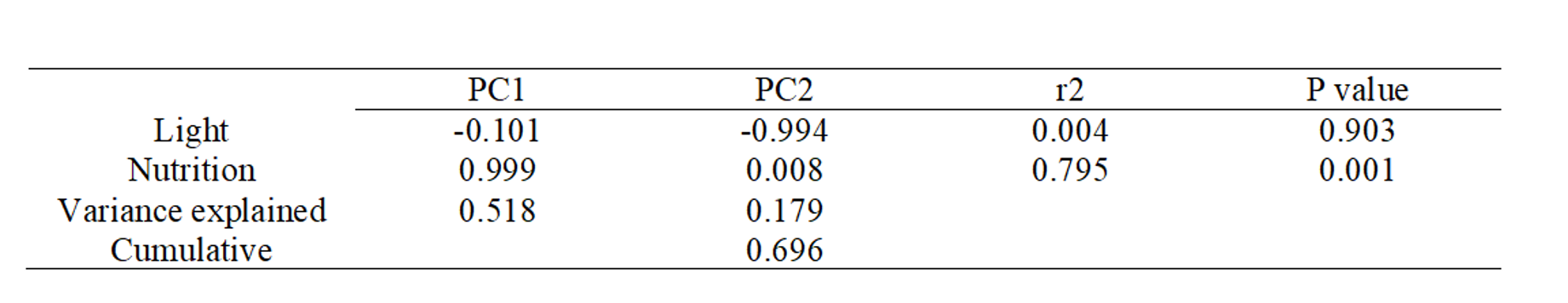

Supplement: S2 Table — (TIF) [file pone.0232144.s007.tif]

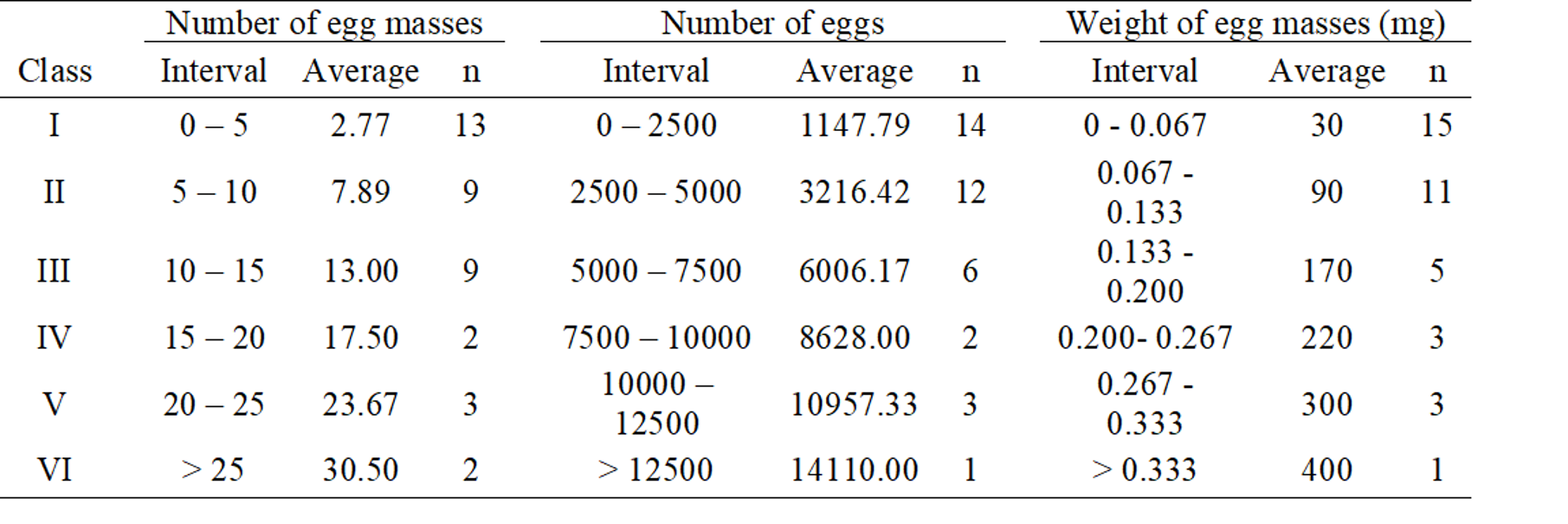

Supplement: S3 Table — (TIF) [file pone.0232144.s008.tif]
