# Supplementary material for: Optimization of Hermetia illucens (L.) egg laying under different nutrition and light conditions
Source: PLoS One. 2020 Apr 24;15(4):e0232144. doi: 10.1371/journal.pone.0232144 (PMC7182258; doi:10.1371/journal.pone.0232144)
Supplement: Attachment — Submitted filename: Response to Reviewers.docx [file pone.0232144.s009.docx]

Dear Academic Editor Humberto Lanz-Mendoza of “PLOS ONE” Journal,

We appreciated very much that our manuscript was considered for publication in your Journal. We really need to thank the editor and the reviewers because thanks to their suggestions we had the chance to perform further analysis that allowed a great improvement of the manuscript, which was rewritten in many parts. Specifically, very interesting results were obtained considering the trade-off between the longevity and reproduction of flies. Please find our rebuttal below.

In the revised version with the tracking of changes, with red it is written the part where the existing information was reorganized, compared to the previous version; highlighted in yellow are the paragraphs that where added, according to the requests of reviewers.

Academic editor suggestions#:

1) The authors require to incorporate longevity in their analysis to ensure that you do not have a trade-off between longevity and reproduction,

2) The authors need to show data regarding egg hatchability and larval survival for the females treated with MSW.

The authors require to incorporate longevity in their analysis to ensure that you do not have a trade-off between longevity and reproduction, and the authors need to show data regarding egg hatchability and larval survival for the females treated with MSW.

All the requested information is described below.

Reviewer #1:

1) Egg Hatchability is a reference to colony efficiency for a mass rearing industrial purpose, and so is the larvae survival. In my opinion in order to conclude that the nutrition and light treatment (MSW) is be the best treatment for industrial purposes, the authors need to show data regarding egg hatchability and larvae survival for the females treated with MSW. They only show data regarding the light but not the nutrition treatment.
   The authors mention that egg hatchability is not influenced by adult nutrition, according to reference 28. However, the mentioned reference does show a positive impact of the nutritional treatment enhancing the hatching performance of H. illucens eggs. Therefore, it is necessary to present egg hatchability results regarding their MSW treatment, and the other treatments, along with the control, since it could be an interesting reference for optimizing rearing methods.
   On the other hand, the percentage of hatchability seems to be low for H. illucens, and the authors do not show a reference nor control data.

We totally agree that providing data on hatchability would have been an interesting reference for optimizing rearing methods. However, verifying the conditions to improve egg hatching was not the purpose of the present study. Certainly this will be the aim of our future research. We rewrote the paragraph and specified this in lines 425-430.

As well, we stated that the MSW treatment registered the highest amount of eggs produced. However, based on data provided by the oviposition rate (80% egg), we recommend the MW treatment for industrial purposes.

Besides, we rewrote many other parts to add specific useful information, as follows:

Line 68-71 we added and highlighted the information: ‘’Egg hatchability seemed to be positively influenced by LED lighting compared to fluorescent tubes (15)while a positive influence was observed by feeding adults with a combination of sugar, milk powder and bacteriological peptone, although the difference was not significant if compared to agar, water or no nutrition (18).”

Line 183-186 we added details: ‘’Considering that light source is a key factor for BSF mating and for obtaining fertilized eggs (23,26) and that no significant differences on egg hatchability have been observed for different adult feeding strategies (18), we assessed the influence of different lights on egg hatching with a single diet treatment (W treatment).”

Line 192-195 we added details: ‘’10 mg of eggs (<24 h old) were collected, weighed on a Petri dish (90 mm diameter) and placed directly on the rearing substrate, consisting in Gainesville House Fly diet (29,30). Controls were performed twice a week, when fresh substrate and tap water were added. After 14 days the larval biomass was removed and counted (15)’’.

Lines 425-430 we added explanation: “The low number of larvae that survived might be due to the negative effects of fungi or other microbial growth as previously reported in literature (14,38), also because in our case the fresh eggs (<24 h) were placed directly on the rearing substrate. In this work the aim was to test the egg hatchability after the flies were exposed to different lights and not to verify the best conditions to increase hatching. Therefore, in the view to improve egg hatching success for the implementation of industrial mass rearing further specific studies are needed.’’

Reviewer #2:

Dear Authors and Editor. First at all my apologist for taking so long reviewing this paper. I was very. Very ill, and just now I take the time to read it.

The paper is intersecting. As a major comment are:

1) a) I propose you to better present your results

Thank you very much for your suggestion, we reorganized all the ‘’Material and methods’’, ’’Results’’ and ‘’Discussion’’ sections. The article now has a better flow and the results are easier to follow.

2) b) incorporate longevity in your analyze just to ensure that you do not have trade-off between longevity and reproduction.

Due to the fact that the overall egg amount and the life span or BSF females and males were already individually analyzed and presented, we introduced a Non-metric analyze for the trade-off between longevity and reproduction of BSF flies, under the influence of nutrition and light, in a graphical way (Fig 4 - 5). A whole paragraph has therefore been added to ‘’Results’’ (lines 244-266).

3) Line 27-30. Please be more specific here. Which gaps?

The sentence has been modified and several issues/elements were added.

Currently lines 51-54: ‘’Although abundant studies were performed on the optimization of larvae rearing (3,14–17), while additional information on the adults breeding strategies, such as individual reproductive potential of females or adult density and nutrition needs must be deepened (18,19), in order to increase egg production and achieve a successful industrial process.’’

4) Line 95-97. Did you test all?

After the changes made, the lines correspond to the numbers 119-122.

Neon tube is the common name of fluorescent lamp. The emission of these type of light source is due to the excitation of different atoms and, therefore, the spectrum emission consists in a series of several specific wavelength or lines (as we measured with a spectrometer).

We did not try different fluorescent light because their spectrum is always due to the same atoms: mercury, terbium and europium. Different mixes of these atoms can produce on the human eye the effect of different colors. In reality, the characteristic wavelengths are the same, and, the color effect is due to the different relative intensity of the atoms.

For the purpose of our experiment we are interested only in the effect of different wavelengths on the BSF, for this reason we can conclude that the effect of the lamp, used in the article, can be representative for all the fluorescent lamp family.

5) Results are very difficult to read. Maybe you can use a figure to explain it graphically. In addition, you may present the PCA first, and then, you can use ANOVAS with more interested results revealed in the PCA. That depends from you. My point is to you present much better your results

We began the ‘’Results’’ presentation by exposing the Life Span of adults and we improved the interpretation of PCA analysis (lines 213-227). Further, starting with Line 244, we continued with the presentation of Trade-off analysis.

6) Discussion, could you compare longevity between treatments. According to life-history theory, reproduction is traded of with longevity: The more eggs, the less longevity.

Please see previous comments at point 2)-b) and we added more discussions in lines 384-395.
